# Downregulation of GLI3 Expression Mediates Chemotherapy Resistance in Acute Myeloid Leukemia

**DOI:** 10.3390/ijms21145084

**Published:** 2020-07-18

**Authors:** Fabian Freisleben, Lena Behrmann, Vanessa Thaden, Jana Muschhammer, Carsten Bokemeyer, Walter Fiedler, Jasmin Wellbrock

**Affiliations:** Department of Oncology, Hematology and Bone Marrow Transplantation with Section Pneumology, Hubertus Wald University Cancer Center, University Medical Center Hamburg-Eppendorf, 20251 Hamburg, Germany; freisleben.fabian@gmail.com (F.F.); le.behrmann@uke.de (L.B.); v.thaden@uke.de (V.T.); j.muschhammer@uke.de (J.M.); c.bokemeyer@uke.de (C.B.); fiedler@uke.de (W.F.)

**Keywords:** AML, GLI3, HH, cytarabine, Ara-C, resistance, *SAMHD1*, *CDA*, *ABCC11*

## Abstract

Aberrant activation of the hedgehog (HH) pathway is observed in many neoplasms, including acute myeloid leukemia (AML). The glioma-associated oncogene homolog (GLI) transcription factors are the main downstream effectors of the HH signaling cascade and are responsible for the proliferation and maintenance of leukemic stem cells, which support chemotherapy resistance and leukemia relapse. Cytarabine (Ara-C)-resistant variants of AML cell lines were established through long-term cultivation with successively increasing Ara-C concentrations. Subsequently, differences in *GLI* expression were analyzed by RT-qPCR. *GLI3* mRNA levels were detectable in parental Kasumi-1, OCI-AML3, and OCI-AML5 cells, whereas *GLI3* expression was completely silenced in all resistant counterparts. Therefore, we generated GLI3-knockdown cell lines using small hairpin RNAs (shRNA) and evaluated their sensitivity to Ara-C in vitro. The knockdown of GLI3 partly abolished the effect of Ara-C on colony formation and induction of apoptosis, indicating that *GLI3* downregulation results in Ara-C resistance. Moreover, we analyzed the expression of several genes involved in Ara-C metabolism and transport. Knockdown of GLI3 resulted in the upregulation of SAM and HD domain-containing protein 1 (*SAMHD1*), cytidine deaminase (*CDA*), and ATP-binding cassette C11 (*ABCC11*)/multidrug resistance-associated protein 8 (*MRP8*), each of which has been identified as a predictive marker for Ara-C response in acute myeloid leukemia. Our results demonstrate that *GLI3* downregulation is a potential mechanism to induce chemotherapy resistance in AML.

## 1. Introduction

Attaining sustained long-term remission in acute myeloid leukemia (AML) patients presents a notable therapeutic challenge. Despite high initial response rates to chemotherapy, the majority of patients suffer from a relapse, ultimately leading to death in most cases [1,2]. Growing evidence indicates that relapse is caused by a small population of leukemic stem cells (LSCs) resistant to chemotherapy, which serve as reservoir for leukemic blasts [3,4]. In the bone marrow niche, hematopoietic stem cells (HSC) maintain their stemness and survival by bidirectional crosstalk with the bone marrow microenvironment [5]. LSCs are able to infiltrate the niche and alter homeostatic processes to maintain their quiescence, survival, and resistance to chemotherapy [6]. The interaction of LSC with the microenvironment involves a variety of stem cell signaling pathways, including the hedgehog (HH) signaling pathway [7].

The HH signaling pathway is a highly conserved signaling cascade that plays a critical role during embryogenesis and is strongly involved in many basic cellular functions, including cell differentiation and proliferation and stem cell maintenance [8]. It is well established that aberrant hedgehog signaling is associated with a wide variety of neoplasms [9], which results in the activation of the GLI transcription factors, the main downstream effectors of the HH signaling cascade. In previous work, we could show that *GLI* expression represents a negative prognostic factor in AML [10].

The GLI transcription factors consist of three members with specialized function and distinct regulation mechanisms: GLI1, GLI2, and GLI3. GLI1 and GLI2 represent transcriptional activators, whereas GLI3 occurs predominantly in its repressor form and functions as a strong repressor of GLI-mediated transcription [11,12,13]. In the canonical HH pathway, SMO regulates the level of GLI activity by shifting the balance between transcriptional stimulation through activated GLI2 and inhibition through GLI3 in its repressor form (GLI3R), while GLI1 is not expressed in resting cells [13,14,15]. However, GLI transcription factors represent central hubs in the oncogenic signaling network and can get activated non-canonically by cross-talk with a variety of pathways, including FLT3, PI3K-AKT, RAS–RAF–MEK, or TGFβ [16,17]. In AML cells, HH activation is largely independent of SMO activity but is strongly suppressed by GLI3R protein expression [18]. Analysis of The Cancer Genome Atlas AML data set has shown that *GLI3* expression is epigenetically silenced in the majority of AML patient samples [18]. Consistent with these findings, we could show that *GLI3* expression is absent in most AML patients as determined by qPCR analysis [10].

Several studies have supported the role of activated GLI signaling in the development of resistance to chemotherapy in multiple cancers, including AML, gastric cancer, and ovarian cancer [19,20,21]. While it has been shown that chemotherapy resistance can be caused by aberrant activation of the transcriptional activators GLI1 or GLI2 [22,23], changes in *GLI3* expression have never been described in this context in AML. We hypothesized that the transcriptional repressor GLI3 may represent a major switch involved in sensitivity to chemotherapy.

## 2. Results

### 2.1. GLI3 Expression Silenced in Cytarabine (Ara-C)-Resistant Cell Lines

**Figure 1:**
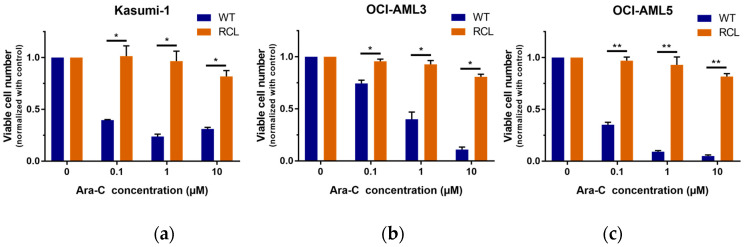
Number of viable cells in resistant vs. parental cell lines following treatment with increasing cytarabine (Ara-C) concentrations. Resistant cell lines (RCL) and wildtype (WT) variants of acute myeloid leukemia (AML) cell lines Kasumi-1 (**a**), OCI-AML3 (**b**), OCI-AML5 (**c**) were plated with different concentrations of Ara-C ranging from 100 nM to 10,000 nM. Cell counts were normalized to the those in untreated controls. The average number of viable cells (Ø) in the untreated control samples was 1.00 × 10^6^ (Kasumi-1), 1.54 × 10^6^ (OCI-AML3), and 1.43 × 10^6^ (OCI-AML3) for WT cells and 1.96 × 10^6^ (Kasumi-1), 1.92 × 10^6^ (OCI-AML3), and 1.75 × 10^6^ (OCI-AML3) for RCL; * *p* < 0.05 and ** *p* < 0.01 in Welch’s *t*-test.

**Figure A1:**
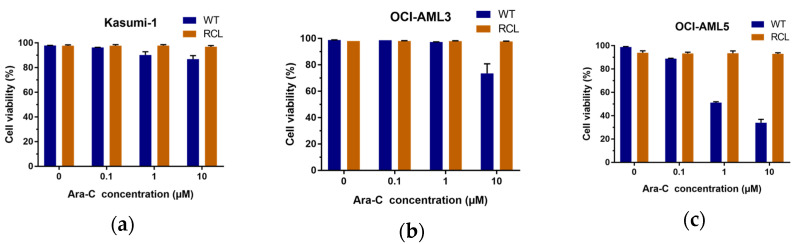
Cell viability of resistant and parental cell lines following treatment with increasing Ara-C concentrations. Resistant (RCL) and wildtype (WT) variants of AML cell lines Kasumi-1 (**a**), OCI-AML3 (**b**), OCI-AML5 (**c**) were plated with different concentrations of Ara-C ranging from 100 nM to 10,000 nM. Cell viability was determined after 3 days with the Trypan Blue dye exclusion method.

To better understand the role of the hedgehog pathway in the development of drug resistance and relapse in AML, we generated Ara-C-resistant variants of the AML cell lines Kasumi-1, OCI-AML3, and OCI-AML5 through long-term cultivation with successively increasing Ara-C concentrations. Ara-C resistance was characterized by an IC80 value (80% inhibitory concentration) for cell growth above 10,000 nM (refer to Figure 1 for relative number of viable cells and Figure A1 for cell viability, respectively).

**Figure 2:**
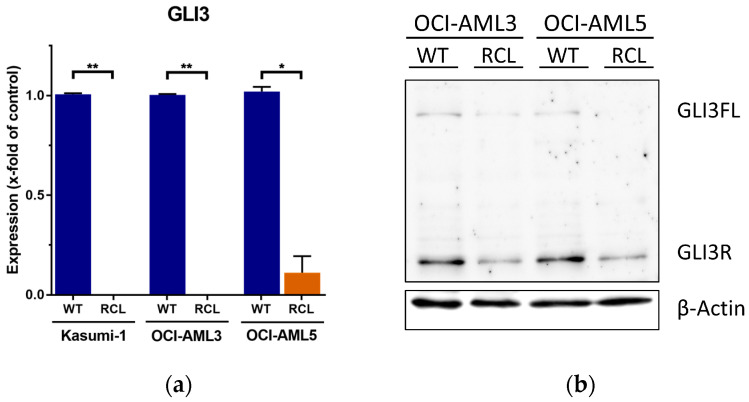
Downregulation of GLI3 in Ara-C resistant cell lines. (**a**) WT and Ara-C-RCL Kasumi-1, OCI-AML3, and OCI-AML5 were analyzed for *GLI3* mRNA levels by RT-qPCR analysis; * *p* < 0.05 and ** *p* < 0.01 in Welch’s *t*-test. (**b**) Western blot of full length GLI3 (GLI3FL) and its repressor form (GLI3R) in WT and RCL variants of OCI-AML3 and OCI-AML5.

**Figure A2:**
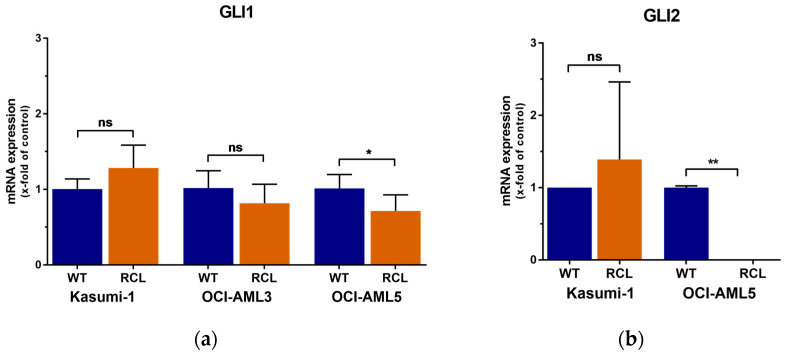
No consistently significant changes in *GLI1* and *GLI2* mRNA expression in Ara-C-resistant cell lines. (**a**) WT and Ara-C RCL Kasumi-1, OCI-AML3, and OCI-AML5 were analyzed for *GLI1* and *GLI*2 mRNA levels by RT-qPCR. Relative *GLI2* expression could not be analyzed for OCI-AML3, as the *GLI2* expression levels were below the detection level. Error bars represent the mean values ± standard deviation; * *p* < 0.05, ** *p* < 0.01 in the Welch’s *t*-test; ns, not significant.

Subsequently, *GLI* expression was analyzed in resistant variants and compared to that in their respective parental cell lines. We could not detect consistently significant changes in *GLI1* and *GLI2* mRNA expression (Appendix A, Figure A2). However, RT-qPCR analysis revealed that *GLI3* expression was completely silenced in Ara-C-RCL Kasumi-1, OCI-AML3, and OCI-AML5, whereas *GLI3* mRNA levels were detectable in their parental counterparts (Figure 2a). Moreover, we could show that resistant OCI-AML3 and OCI-AML5 cells had lower protein levels of both full length GLI3 and its repressor form compared to their parental cell line, using western blot analysis (Figure 2b).

### 2.2. GLI3 Knockdown Promotes Resistance to Chemotherapy

**Figure 3:**
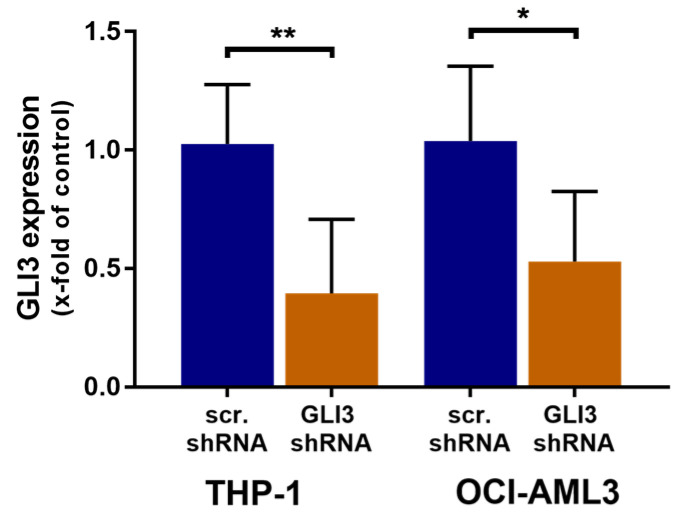
Efficiency of GLI3 knockdown in AML cell lines THP-1 and OCI-AML3. *GLI3* mRNA levels were measured by RT-qPCR following lentiviral transduction with two distinct shRNA targeting *GLI3*. The expression of *GLI3* was normalized to that in control cells transduced with a scrambled control shRNA; * *p* < 0.05 and ** *p* < 0.01 in Welch’s *t*-test.

**Figure A3:**
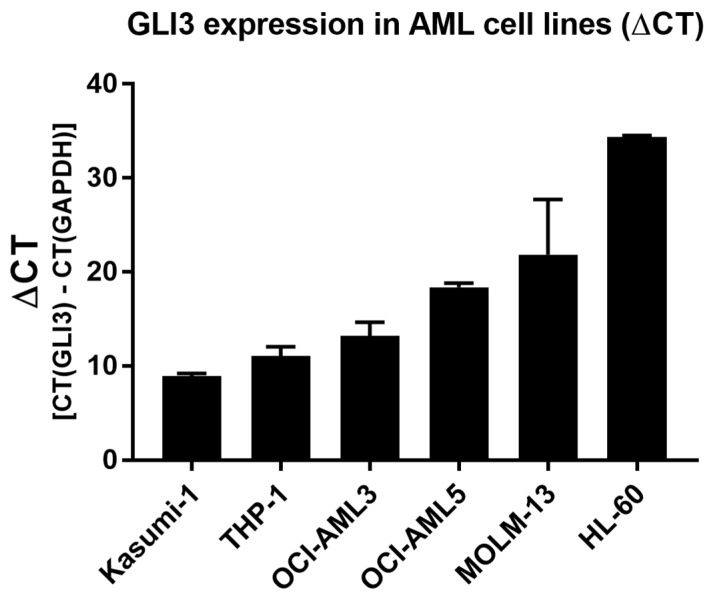
Expression levels of *GLI3* in different AML cell lines. *GLI3* expression in AML cell lines Kasumi-1, THP-1, OCI-AML3, OCI-AML5, MOLM-13, and HL-60 was quantified by RT-qPCR. ΔCT values were calculated with the following formula: *ΔCT = CT(GLI3) −CT(GAPDH)*. Small ΔCT values indicate high *GLI3* expression. If no expression was found, the CT values were equated with the total cycle number (50 cycles). No *GLI3* expression was found in HL-60 cells. Error bars represent the mean values ± standard deviation.

For shRNA experiments, we chose the AML cell lines THP-1 and OCI-AML3 that express the highest levels of *GLI3* (Figure A3). To investigate whether GLI3 silencing alone imparted drug resistance, we generated GLI3-knockdown cells by lentiviral transduction of two distinct *GLI3*-specific shRNAs. AML cells with GLI3 knockdown were compared with control cells containing nontargeting shRNA. Compared with the control, *GLI3* expression was reduced to 39.6% (± 31.1%) in THP-1 cells and to 53.7% (± 31.4%) in OCI-AML3 cells (Figure 3).

**Figure 4:**
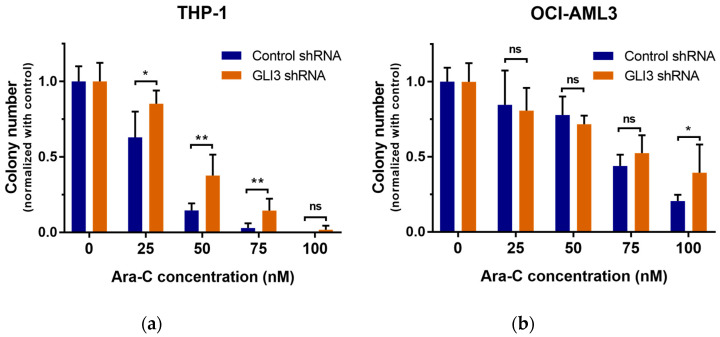
GLI3 knockdown partially protects AML cells against the cytotoxic effect of Ara-C. AML cell lines THP-1 (**a**) and OCI-AML3 (**b**) after GLI3 knockdown and treatment with scrambled shRNA control were subjected to different Ara-C concentrations for 7 days. Colony numbers were counted and normalized to those of the untreated controls. The average number of colonies (Ø) in the untreated control samples was 84 (scrambled shRNA) and 62 (GLI3 shRNA) for THP-1 and 72 (scrambled shRNA) and 83 (GLI3 shRNA) for OCI-AML3; * *p* < 0.05 and ** *p* < 0.01 in Welch’s *t*-test; ns, statistically not significant.

We performed colony formation assays to investigate whether GLI3 knockdown affects the ability of leukemic cells to form colonies upon exposure to Ara-C. GLI3-knockdown and scrambled shRNA control AML cell lines THP-1 and OCI-AML3 were treated with Ara-C concentrations ranging from 25 to 100 nM. Colony numbers were counted on day 7 and normalized to the untreated control. For both GLI3-knockdown cell lines, Ara-C treatment reduced the colony numbers significantly compared to the control cells (Figure 4a,b).

**Figure 5:**
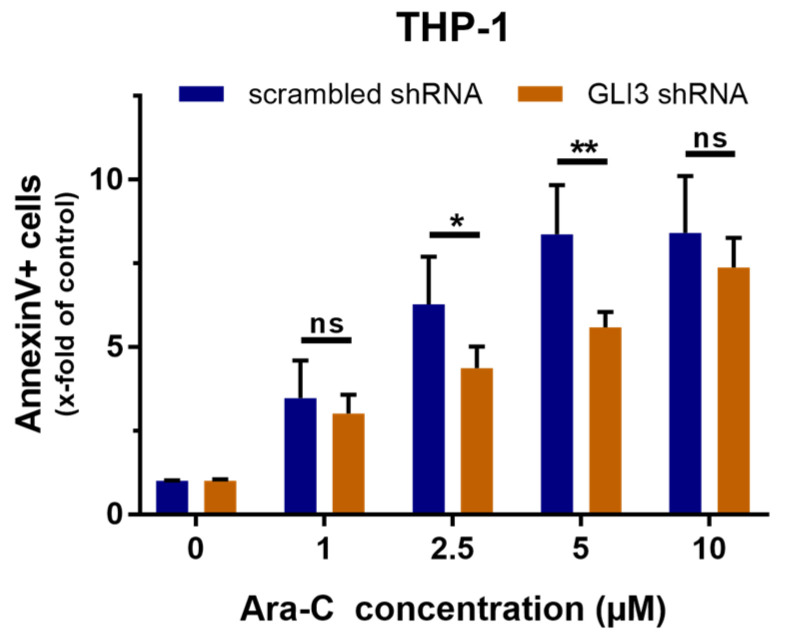
GLI3 knockdown suppresses apoptosis induction upon treatment with Ara-C. THP-1 cells transduced with GLI3-targeted shRNA or scrambled shRNA control were treated with the indicated concentrations of Ara-C, and induction of apoptosis was measured after 48 h by flow cytometry using Annexin V and propidium iodide. In the untreated (control) samples, the majority of cells transduced with scrambled shRNA (Ø 5.9% Annexin V-positive) or GLI3 shRNA (Ø 6.9% Annexin V-positive) were viable. Representative flow cytometry plots are shown in Figure A4 (Appendix A). Error bars represent the mean values ± standard deviation; * *p* < 0.05, ** *p* < 0.01 in the Welch’s *t*-test; ns, statistically not significant.

THP-1 cells transduced with either GLI3-targeted shRNA or non-targeting control shRNA were investigated for apoptosis induction upon Ara-C treatment. Cells were treated with Ara-C concentration ranging from 1 µM to 10 µM, and apoptosis rates were determined by flow cytometry. GLI3 knockdown had the most pronounced effect on apoptosis rates in the presence of high concentrations of Ara-C, with significant differences observed at 2.5 and 5 µM (Figure 5).

### 2.3. GLI3 Knockdown Impacts the Expression of Ara-C Resistance Genes

**Figure 6:**
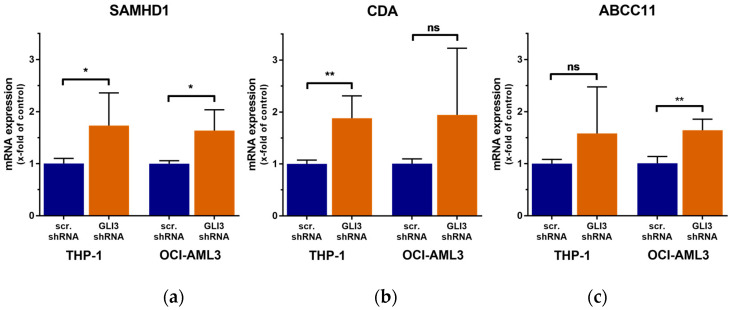
Knockdown of GLI3 results in the upregulation of genes involved in Ara-C metabolism and transport. Expression levels of *SAMHD1* (**a**), *CDA* (**b**), and *ABCC11* (**c**) in GLI3-knockdown cell lines THP-1 and OCI-AML3 were quantified by RT-qPCR and compared to their expression levels in control cells transduced with a scrambled shRNA. Error bars represent the mean values ± standard deviation; * *p* < 0.05, ** *p* < 0.01 in the Welch’s *t*-test; ns, not significant.

Because GLI3 knockdown reduced the sensitivity of AML cells to Ara-C, we next assessed whether gene knockdown was associated with expression changes of several genes involved in Ara-C metabolism and transport. RT-qPCR analysis revealed that *SAMHD1*, *CDA*, and *ABCC11* (*MRP8*) were upregulated in GLI3-knockdown cells compared to cells transduced with control shRNA (Figure 6).

**Figure 7:**
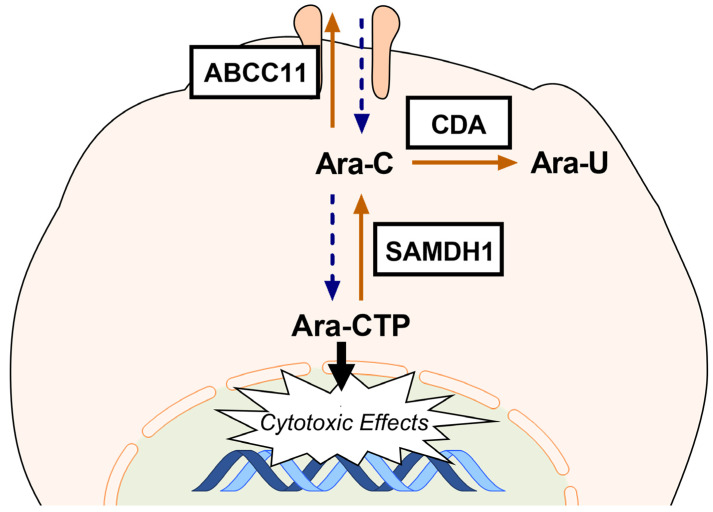
Role of SAMHD1, CDA, and ABCC11 in Ara-C metabolism. ATP-binding cassette C11 (ABCC11) functions as a nucleotide efflux pump and reduces the intracellular levels of Ara-C; cytidine deaminase (CDA) irreversibly deaminates Ara-C to its inactive uracil derivative uracil arabinoside (Ara-U); the phosphohydrolase SAMHD1 reduces active Ara-CTP levels through hydrolyzing Ara-CTP into inactive Ara-C.

SAMHD1 and CDA are two key enzymes of the Ara-C metabolism that strongly reduce the intracellular level of the active Ara-C metabolite by promoting the conversion of Ara-C to an inactive state. In addition, GLI3 knockdown increased the expression of ABCC11, a membrane transporter with the ability to efflux nucleoside analogues, such as Ara-C, inhibiting their intracellular accumulation (Figure 7).

## 3. Discussion

Despite high responses to initial chemotherapy, the vast majority of AML patients relapses after remission due to persistent subpopulation of LSC, because of their drug-resistant phenotype. The LSC hypothesis is of substantial clinical relevance, offering an explanation for minimal residual disease, relapse, and therapy failure and highlighting the need to target these cells in order to achieve long-lasting remissions [24,25].

In mammals, three GLI transcription factors function as central mediators of HH signaling. GLI1 only functions as a transcriptional activator [26], while GLI2 and GLI3 can function both as activating and as inhibitory regulators [27]. Full-length GLI3 (GLI3FL), after phosphorylation and nuclear translocation, acts as a weak transcriptional activator [28]. The proteolytically processing of GLI2FL to its repressor form is not present in cultured cell lines or, at best, is inefficient. The majority of GLI2FL is degraded completely by the proteasome. In contrast, GLI3FL is efficiently processed to the truncated GLI3-repressor form that acts as a strong negative regulator of GLI-mediated transcription [11]. In the absence of HH signaling, GLI3 is predominantly in its repressor form and functions as a strong repressor of GLI-mediated transcription. The level of GLI signaling activity is largely determined by the balance between the transcriptional activators GLI1 and GLI2 and the repressor GLI3R [14,15]. 

To investigate the molecular changes underlying resistance to chemotherapy, we generated Ara-C-resistant strains of several AML cell lines and performed gene expression analysis of the HH pathway members using RT-qPCR and western blot. We showed that *GLI3* expression was silenced in AML cells with acquired Ara-C resistance. A tumor suppressor role for GLI3R has been demonstrated in a medulloblastoma mouse model driven by GLI2ΔN expression. GLI2ΔN is a constitutively active GLI2 isoform. In the absence of cilia, GLI2ΔN induces medulloblastoma early in life by elimination of GLI3R [29]. The primary cilium is required for proteolytical processing of GLI3 to its repressor form [30]. Interestingly, it has been shown that primary cilia are absent in a high proportion of AML cells, possibly resulting in reduced intracellular GLI3R levels in most cases [31]. In line with this hypothesis, we could observe the absence of *GLI3* expression in 74% of AML patient samples [10]. Consistent with these results, genetic analysis of The Cancer Genome Atlas AML dataset by Chaudhry et al. demonstrated that *GLI3* expression is epigenetically silenced in most AML patients [18]. In agreement with these findings, when analyzing AML cell lines by RT-qPCR, *GLI3* expression could not be detected in HL-60 cells, while low expression was found in MOLM-13 and OCI-AML5 AML cell lines. The highest *GLI3* mRNA levels could be detected in Kasumi-1, THP-1, and OCI-AML3 cells (Appendix A, Figure A3). As previously mentioned, *GLI3* is epigenetically silenced in a large number of AML cells, which suggests that *GLI3* expression was also downregulated through epigenetic mechanisms in the Ara-C-resistant subclones. Accordingly, it has been demonstrated that *GLI3* expression could be restored in AML cells treated with decitabine, a hypomethylating agent [18].

We showed that downregulation of *GLI3* using shRNA reduced cell sensitivity towards Ara-C treatment. This effect was especially obvious in clonogenic assays of AML cells. This indicates that *GLI3* downregulation might specifically protect leukemic stem or progenitor cells from the cytotoxic effects of Ara-C. Even though GLI3 silencing has never been described in the context of Ara-C resistance in AML, the association of HH pathway activity with chemotherapy resistance is well established in leukemia and other cancers. Queiroz and colleagues showed that activation of the HH pathway was associated with a multidrug-resistant phenotype of myeloid leukemia cells by upregulation of *p*-glycoprotein, a drug efflux pump [32]. Several studies demonstrated that the combination of Ara-C with the SMO inhibitor cyclopamine or the GLI inhibitor GANT-61 significantly enhanced the sensitivity of AML cell lines and primary CD34^+^ AML cells to Ara-C [21,33,34]. In a recent study, *GLI1* expression was significantly higher in refractory patients compared to non-refractory cases. In addition, high expression of *GLI1* was associated with rapid and repeated relapse. The authors could reverse resistance in the multiple drug-resistant HL-60 AML cell line using the SMO inhibitor NVP-LDE225, resulting in decreased protein expression of MRP1, which is a membrane drug transporter protein responsible for drug resistance and a poor prognosis in AML patients [35]. Furthermore, activated GLI signaling results in the upregulation of several drug transporters, including the ABC transporters *ABCB1*, *ABCB2*, and *ABCG2*, DNA repair mechanisms, and drug-modifying enzymes of the UDP glucuronosyltransferase (*UGT1A*) family [22,36,37,38]. However, while the role of GLI signaling in drug resistance is well established, the involvement of *GLI3* gene expression in the development of chemotherapy resistance has not been investigated.

We could show that GLI3 downregulation resulted in increased expression of *SAMHD1*, *CDA*, and *ABCC11* (*MRP8*). ATP-binding cassette C11 (*ABCC11*) is a member of the multidrug resistance-associated protein (MRP) family of ATP-binding cassette transporters, which functions as a nucleotide efflux pump and has been shown to reduce the intracellular levels of several clinically relevant nucleotide analogs, including the anticancer fluoropyrimidines and antiviral agents [39]. The expression of the efflux transporter *ABCC11* correlates with poor prognosis in AML. Cells transfected with ABCC11 were resistant to Ara-C and showed reduced intracellular levels of Ara-C and its metabolites [40]. Intracellularly, Ara-C is activated through three phosphorylation steps leading to its active metabolite cytidine-5′-triphosphate (Ara-CTP), with phosphorylation of Ara-C to Ara-CMP by deoxycytidine kinase (DCK) being the rate-limiting step in its activation [41]. *SAMHD1* is a phosphohydrolase that cleaves deoxynucleoside triphosphates (dNTP) into inorganic triphosphate and deoxyribonucleosides [42]. In leukemic cells exposed to Ara-C, SAMHD1 drastically reduces Ara-CTP levels through hydrolyzing Ara-CTP into inactive Ara-C [43]. Schneider et al. showed that inactivation of SAMHD1 strongly sensitizes AML cells to the cytotoxic effects of Ara-C in vitro and in vivo. Moreover, they showed that *SAMHD1* expression is a negative predictor of the response to Ara-C-based treatment in AML patients [44]. In the activation of Ara-C, DCK competes with cytidine deaminase (CDA), which irreversibly deaminates Ara-C to its inactive uracil derivative uracil arabinoside (Ara-U) [45]. Ohta et al. showed that high CDA activity mediates the resistance of U937 monocytoid leukemia cells to Ara-C [46]. In an ex vivo cytotoxicity assay of AML patient samples, *CDA* expression was significantly lower in the Ara-C-sensitive group compared with intermediately sensitive or resistant samples and was found to be a strong predictor of Ara-C response [47]. In AML patients, high activity and expression of *CDA* was associated with poor initial response and predictive of remission duration [48,49].

In conclusion, we describe that loss of GLI3R through *GLI3* gene silencing in AML cells results in acquired Ara-C resistance. GLI3R functions as a strong repressor of GLI-mediated transcription, and its downregulation by shRNA significantly reduces the effect of Ara-C in AML cells by modulating key enzymes involved in Ara-C metabolism.

## 4. Materials and Methods 

### 4.1. Cell Lines and Cell Culture

The cell lines used in this study were either purchased from the DSMZ (Deutsche Sammlung von Mikroorganismen und Zellkulturen GmbH, Braunschweig, Germany) or authenticated by the Multiplex human Cell Authentication test (Multiplexion GmbH, Heidelberg, Germany). THP1 cells were maintained in RPMI 1640 medium (Gibco, Thermo Fisher Scientific, Waltham, MA, USA) supplemented with 10% fetal bovine serum (FBS Superior, Biochrom GmbH, Berlin, Germany). Kasumi-1 cells were cultured in RPMI 1640 medium supplemented with 20% FBS. OCI-AML3 cells were maintained in α-MEM medium (Gibco, Thermo Fisher Scientific, Waltham, MA, USA) supplemented with 20 % FBS. OCI-AML5 cells were cultured in α-MEM mediumsupplemented with 20 % FBS and 10 ng/mL GM-CSF (PeproTech GmbH, Hamburg, Germany). All cells were maintained in a humidified incubator with 5 % CO_2_ at 37 °C.

### 4.2. Generation of Ara-C-Resistant Cell Lines

Kasumi-1, OCI-AML3, and OCI-AML5 cells were cultivated with their respective IC95 Ara-C concentration continuously for several months. Cell viability was measured twice a week on day 3 and day 7, and Ara-C dose was adjusted according to cellular IC95 rates. Cells were routinely tested for Ara-C resistance in proliferation assays with Ara-C concentration up to 10,000 nM. Resistance was defined as IC80 > 10,000 nM Ara-C. All cell lines (resistant and parental) were routinely checked to ensure there was no mycoplasma contamination, using MycoAlert Mycoplasma Detection kit (Lonza Group AG, Basel, Swiss).

### 4.3. Lentiviral Transduction of AML Cell Lines with GLI3-Specific shRNA

Two different pLKO.1-puro vectors encoding GLI3 (#1, TRCN0000416117, sequence 5′-CCGGACAAGAGGTCCAAGATCAAACCTCGAGGTTTGATCTTGGACCTCTTGTTTTTTTG-3′ and #2, TRCN0000020506, sequence 5′-CCGGGCCATCCACATGGAATATCTTCTCGAGAAGATATTCCATGTGGATGGCTTTTT-3′) or scrambled shRNA (SHC002, non-target shRNA vector) were purchased from Sigma-Aldrich (Taufkirchen, Germany). We used the Lentiviral Gene Ontology Vector (LeGO) system for cloning and transfection into the AML cell lines (LeGO-C/Zeo and LeGO-G/Puro, respectively) [50]. Lentiviral particle-containing supernatants were generated in HEK293T cells co-transfected with the plasmids LeGO-C/Zeo + GLI3 shRNA (#1), LeGO-G/Puro + GLI3 shRNA (#2), or LeGO-G/Puro + scrambled shRNA in combination with pMD2.G-VSV-G and psPAX2-Gag-Pol, using calcium phosphate co-precipitation. THP-1 or OCI-AML3 were transduced either with non-targeting shRNA (negative control) or with two shRNA against *GLI3*, simultaneously. On day 3 after transduction, the transduced cells were selected by treatment with puromycin (2 μg/mL; Sigma-Aldrich, Taufkirchen, Germany) and/or zeocin (500 µg/mL; Thermo Fisher Scientific, Waltham, MA, USA) for 7 days prior to functional assays. The knock-down efficiency for *GLI3* was determined using quantitative PCR analysis after 7 days of zeocin and/or puromycin selection. All work with lentiviral particles was done in an S2 facility after approval according to German law. 

### 4.4. Proliferation Assay

Ara-C-resistant and parental cells of the AML cell lines Kasumi-1, OCI-AML3, and OCI-AML5 were plated in 24-well plates at a density of 150,000 cells/well in 500 µL of cell culture medium and cultured with increasing concentrations of Ara-C for 3 days. The number of viable cell was determined after 3 days with the Trypan Blue dye exclusion method, using the cell viability analyzer Vi-Cell ™ XR (Beckman Coulter, Brea, CA, USA).

### 4.5. Apoptosis Assay

THP-1 cells were seeded in 96-well plates at a density of 200,000 cells/well in 200 µL of cell culture medium and incubated with increasing concentrations of Ara-C for 48 h. For shRNA experiments, *GLI3*-knockdown AML cells were compared to the negative control containing non-targeting shRNA. Induction of apoptosis was measured after 48 h by flow cytometry using APC (allophycocyanin)-conjugated Annexin-V (MabTag GmbH, Friesoythe, Germany) and propidium iodide. Data analysis was performed using the FACS Calibur (BD Biosciences, San Jose, CA, USA) and FlowJo X (Version 10.0.7, BD Life Sciences, FlowJo, LLC, Ashland, OR, USA) Software.

### 4.6. Colony Formation Assay

Cell lines were seeded in cell culture dishes (35 × 10 mm, Sarstedt AG & Co. KG, Nümbrecht, Germany) at a density of 250 cells/mL in 1 mL of methylcellulose-based semi-solid medium (Methocult H4230, Stemcell Technologies, Vancouver, BC, Canada) supplemented with different concentrations of Ara-C. For shRNA experiments, the colony formation capacity of AML cell lines with *GLI3* knockdown were compared to that of the negative controls containing non-targeting shRNA. After 7 days, the number of colonies was counted using an inverted microscope (Axiovert 25, Zeiss, Jena, Germany). 

### 4.7. Protein Isolation and Western Blot Analysis

Proteins of OCI-AML3 and OCI-AML5 cells were extracted using the trichloroacetic acid method. Protein concentration was determined using the DC Protein Assay (Bio-Rad Laboratories, Inc., Hercules, CA, USA). For each sample, a total of 20 μg of protein was separated using a 4–12% tris-glycine SDS-polyacrylamide gel (Thermo Fisher Scientific, Waltham, MA, USA). The proteins were transferred to a nitrocellulose membrane, and the membrane was incubated with either polyclonal goat IgG anti-human/mouse GLI3 (AF3690, 1:2000, R&D Systems) or mouse anti-human β-ACTIN (sc-47778, 1:5000, Santa Cruz Biotechnology, Dallas, TX, USA) at 4 °C overnight. HRP-linked anti-goat immunoglobulins (P0449, 1:10,000) and anti-mouse IgG (NXA931, 1:10,000) secondary antibodies were purchased from Dako (Glostrup, Denmark) and GE Healthcare (Chicago, IL, USA), respectively. Membranes were incubated with secondary antibodies for 1 h at room temperature. Imaging was performed using the Amersham ECL Prime Western Blotting Detection Reagent (GE Healthcare, Chicago, IL, USA) and the Fusion SL 4 3500 WL chemiluminescence system (Vilber Lourmat, Eberhardzell, Germany).

### 4.8. Reverse Transcription and Quantitative PCR

Exon-spanning primers were designed with Primer 3 software (Whitehead Institute for Biomedical Research, Boston, MA, USA) or obtained from the GETPrime qPCR primer database [51]. RNA was extracted using innuPREP RNA Mini Kit 2.0 (Analytik Jena, Jena, Germany) and reverse-transcribed into cDNA using PrimeScript™ RT Master Mix (TaKaRa Bio Inc., Kusatsu, Japan). RT-qPCR analyses were carried out on the LightCycler 1.2 (Roche, Basel, Swiss) using the TB Green Premix Ex Taq II (TaKaRa Bio Inc., Kusatsu, Japan) over 40 PCR cycles. The relative expression of the target genes was normalized to that of the reference gene glyceraldehyde 3-phosphate dehydrogenase (*GAPDH*) and calculated using the Pfaffl method [52]. Primers are listed in Appendix A (Table A1).

### 4.9. Statistical Analysis

Data from the in vitro assays were statistically analyzed by the Welch’s *t*-test using GraphPad Prism 7 (GraphPad Software, Inc., San Diego, CA, USA). A *p* value < 0.05 was considered to be statistically significant.

## Figures and Tables

**Figure A4:**
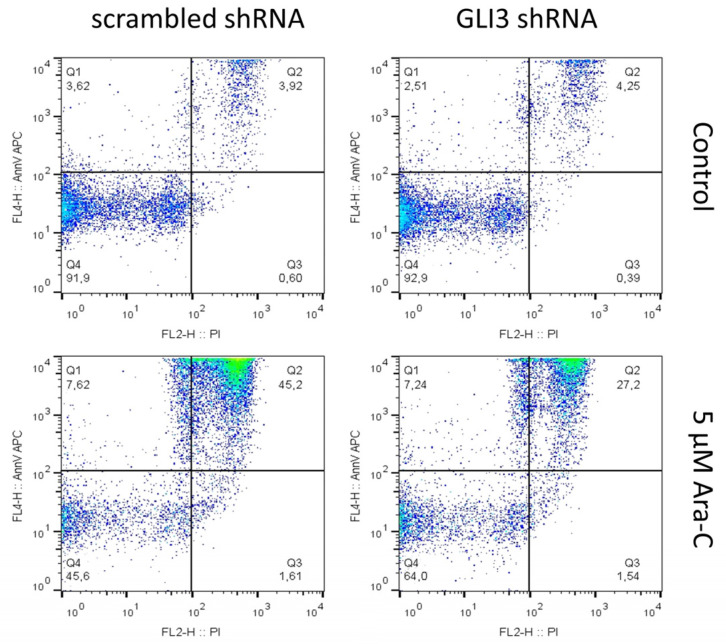
Representative flow cytometry plots for the treatment of THP-1 cells transduced with *GLI3*-targeted shRNA or scrambled shRNA control with 5 µM Ara-C. Induction of apoptosis was measured after 48 h by flow cytometry using Annexin V-APC and propidium iodide.

## Abbreviations

AML	acute myeloid leukemia
LSC	leukemic stem cells
HSC	hematopoietic stem cells
HH	hedgehog (signaling pathway)
GLI	glioma-associated oncogene homolog family zinc finger protein
GLI3FL	GLI3, full length
GLI3R	GLI3, repressor form
Ara-C	cytarabine, cytosine arabinoside
Ara-U	uracil arabinoside
Ara-CTP	aracytidine-5′-triphosphate
SAMHD1	SAM and HD domain-containing protein 1
CDA	cytidine deaminase
ABCC11	ATP-binding cassette C11
MRP8	multidrug resistance-associated protein 8
WT	wildtype
RCL	resistant cell line
shRNA	small hairpin RNA
APC	allophycocyanin

## Appendix A

**Table A1:** Primers used in this study.

Gene	Sense	Anti-Sense
GAPDH	GTCAGTGGTGGACCTGACCT	TGCTGTAGCCAAATTCGTTG
GLI1	CTACATCAACTCCGGCCAAT	CGGCTGACAGTATAGGCAGA
GLI2	GGCCATCCACATGGAATATC	TGAAGAGCTGCTACGGGAAT
GLI3	GGCCATCCACATGGAATATC	TGAAGAGCTGCTACGGGAAT
SAMHD1	ATTGAAAGACGCACGAGAG	AAGAGATTCATAGTCCTCCCT
CDA	GAGAATCTTCAAAGGGTGCA	TTGTACCCTTCTGAGACGG
ABCC11	CCTACTTCATTATTGGATACACTGC	CTTGTCATGAATACCGCCAG
